# Taurine Chloramine-Mediated Nrf2 Activation and HO-1 Induction Confer Protective Effects in Astrocytes

**DOI:** 10.3390/antiox13020169

**Published:** 2024-01-29

**Authors:** Song-I Seol, In Soon Kang, Ji Seok Lee, Ja-Kyeong Lee, Chaekyun Kim

**Affiliations:** 1 Department of Anatomy, Inha University School of Medicine, Incheon 22212, Republic of Korea; 22192191@inha.edu; 2 Laboratory of Leukocyte Signaling Research, Department of Pharmacology, Inha University School of Medicine, Incheon 22212, Republic of Korea; round001@inha.ac.kr (I.S.K.); jiseok@inha.edu (J.S.L.); 3 BK21, Program in Biomedical Science & Engineering, Inha University, Incheon 22212, Republic of Korea

**Keywords:** astrocytes, myeloperoxidase (MPO), nuclear factor E2-related factor (Nrf2), heme oxygenase 1 (HO-1), Kelch-like ECH-associated protein 1 (Keap1)

## Abstract

Taurine is ubiquitously distributed in mammalian tissues, with the highest levels in the brain, heart, and leukocytes. Taurine reacts with hypochlorous acid (HOCl) to produce taurine chloramine (Tau-Cl) via the myeloperoxidase (MPO) system. In this study, we elucidated the antioxidative and protective effects of Tau-Cl in astrocytes. Tau-Cl increased the expression and nuclear translocation of nuclear factor E2-related factor (Nrf2) and the expression of Nrf2-regulated antioxidant genes, including heme oxygenase 1 (HO-1). Nrf2 activity is negatively regulated by Kelch-like ECH-associated protein 1 (Keap1). Tau-Cl decreased the level of the reduced thiol groups of Keap1, resulting in the disruption of the Keap1-Nrf2 complex. Consequently, Tau-Cl rescued the H_2_O_2_-induced cell death by enhancing HO-1 expression and suppressing reactive oxygen species. In conclusion, Tau-Cl confers protective effects in astrocytes by disrupting the Keap1-Nrf2 complex, thereby promoting Nrf2 translocation to the nucleus, wherein it binds to the antioxidant response element (ARE) and accelerates the transcription of antioxidant genes. Therefore, in astrocytes, the activation of the Keap1-Nrf2-ARE pathway by Tau-Cl may increase antioxidants and anti-inflammatory mediators as well as other cytoprotective proteins, conferring protection against brain infection and injury.

## 1. Introduction

Taurine (2-aminoethansulfolic acid) is one of the most abundant nonproteinogenic amino acids; it is ubiquitously distributed in mammalian tissues, with the highest levels found in the heart, brain, and leukocytes [1]. Taurine concentrations range from 1 to 20 μmol/g in the brain and from 20 to 50 mM in human neutrophils [2,3,4]. It plays a vital role in many biological processes, including central nervous system (CNS) and retinal development, membrane stabilization, calcium mobilization, neurotransmission, reproduction, and detoxification [1,5,6]. Furthermore, taurine confers protective effects on infections and inflammation [6,7]. It reacts with the highly toxic hypochlorous acid (HOCl), which is released by the myeloperoxidase (MPO) system of leukocytes, to generate the less toxic taurine chloramine (Tau-Cl) [8]. Tau-Cl inhibits the production of proinflammatory mediators and increases the expression of several antioxidant enzymes. Therefore, taurine ameliorates inflammation by eliminating the highly toxic HOCl as well as modulating inflammatory mediators via Tau-Cl action [5,6].

The brain contains high concentrations of taurine, which functions as an osmoregulator and a neuromodulator [9,10]. Taurine exhibits neuroprotective effects in neurons and astrocytes and animal models of neurological disorders such as ischemic stroke and inflammation [11,12,13,14,15,16]. Furthermore, it decreases the expression of apoptotic protein during ischemic injury [12,17] and maintains intracellular Ca^2+^ homeostasis [18], thereby attenuating apoptotic neuronal death [12,19]. Under pathological conditions, neutrophils infiltrate the regions of inflammatory areas or infected tissues. Neutrophils are the most abundant leukocytes and contain high concentrations of MPO and taurine; therefore, activated neutrophils produce considerable amounts of Tau-Cl. In our previous study, we demonstrated that neutrophils infiltrate ischemic injury areas and that Tau-Cl exerts robust neuroprotective effects using a rat middle cerebral artery occlusion (MCAO) model [20]. Tau-Cl significantly decreased the infarct volume and neurological deficits and promoted motor function. Furthermore, it significantly increased the levels of antioxidant enzymes such as heme oxygenase-1 (HO-1), NAD(P)H:quinone oxidoreductase 1 (NQO1), glutamate–cysteine ligase catalytic (GCLC), and peroxiredoxin-1 (Prx-1) in the post-ischemic brain and in BV2 cells, a microglial cell line [20].

Astrocytes are the most abundant glial cell type in the CNS that outnumbers neurons [21]. Astrocytes perform various functions in the normal brain, including maintaining a stable extracellular environment by regulating the balance of ions and fluid, which are essential for neurons [22]. Furthermore, they control the energy supply to the neurons [23,24] and regulate synapse formation and neurotransmitter turnover [25,26]. Astrocytes are the primary protective cells in the brain. Under pathological conditions, astrocytes confer neuroprotection by regulating ionic balance and energy metabolism and by inducing scar formation, which protects adjacent neural tissues via separating brain injury-induced lesions [21].

Nuclear factor E2-related factor (Nrf2) is a basic-region leucine zipper transcription factor that plays a vital role in oxidative stress response. Nrf2 binds to the antioxidant response element (ARE) located in the promoter regions of several antioxidant and detoxifying genes, including HO-1, NQO1, glutathione S-transferases (GST), and GCL modifier (GCLM) [27,28,29,30,31]. Under basal conditions, Nrf2 activity is tightly regulated by Kelch-like ECH-associated protein 1 (Keap1), an E3 ubiquitin ligase substrate adaptor [32,33]. Keap1 targets Nrf2 for proteasomal degradation. However, during oxidative or electrophilic stress, Keap1 is inactivated by the modification of its highly reactive cysteine residues; as a result, Nrf2 escapes ubiquitination, accumulates within the cell, and translocates to the nucleus, where it promotes its antioxidant transcription program [34,35,36]. Tau-Cl increases the cytosolic accumulation and nuclear translocation of Nrf2, resulting in the high expression of several Nrf2-regulated antioxidant enzymes [20,37,38,39,40]. However, to the best of our knowledge, the mechanism by which Tau-Cl regulates Nrf2 activation remains unclear. 

In this study, we investigated the antioxidant effects of Tau-Cl in astrocytes C6 cells. We determined that Tau-Cl regulates the expression and nuclear translocation of Nrf2 and the subsequent expression of Nrf2-induced genes in C6 cells. Furthermore, we examined the molecular mechanism by which Tau-Cl triggers Nrf2 activation. Ultimately, we explored the protective effects exerted by Tau-Cl on astrocytes. 

## 2. Materials and Methods

### 2.1. C6 Cell Culture and Tau-Cl Treatment

C6 astroglioma cells (American Type Culture Collection, Manassas, VA, USA) were grown in Dulbecco’s modified Eagle’s medium (DMEM; Sigma, St. Louis, MO, USA) supplemented with 5% fetal bovine serum (FBS; Hyclone, Logan, UT, USA), 1% penicillin, and streptomycin at 37 °C in a 5% CO_2_ incubator. Cells were prepared a day before Tau-Cl treatment. Tau-Cl was synthesized by adding equimolar amounts of NaOCl (Aldrich Chemical, Milwaukee, MI, USA) to taurine (Sigma), and the formation of Tau-Cl was monitored by measuring UV absorption (200~400 nm) [41]. Tau-Cl was diluted to 200 μM in DMEM containing 10% FBS. C6 cells were treated with Tau-Cl for the indicated time points. 

### 2.2. Nuclear and Cytoplasmic Extract Preparation 

C6 cells were lysed with solution A (0.5% Triton X-100, 0.5% NP-40, 10 mM HEPES [pH 7.9], 10 mM KCl, 0.1 mM EDTA, and 1 mM DTT) containing complete Mini Protease Inhibitor Cocktail tablet (Roche diagnostics, Basel, Switzerland). Lysates were centrifuged at 17,500× *g* for 5 min at 4 °C. The supernatant containing cytoplasmic protein was collected and stored at −80 °C. Pellets were lysed with solution B (10% glycerol, 20 mM HEPES [pH 7.9], 0.4 M NaCl, 1 mM EDTA, and 1 mM DTT) containing complete Mini Protease Inhibitor Cocktail tablet. Lysates were centrifuged at 17,500× *g* for 10 min at 4 °C. The supernatant containing the nuclear protein was collected and stored at −80 °C.

### 2.3. Immunoblotting

Cells were lysed with RIPA buffer (0.25% sodium-deoxycholate, 150 mM NaCl, 50 mM Tris-HCl [pH 7.4], and 1% NP-40) containing complete Mini Protease Inhibitor Cocktail tablet. Lysates were centrifuged at 12,000× *g* for 10 min at 4 °C and the supernatants were loaded into 12% SDS-PAGE gels. The primary antibodies used were as follows: anti-Nrf2 (sc-722, 1:1000; Santa Cruz Biotechnology, Santa Cruz, CA, USA), anti-α-tubulin (GTX112141, 1:10,000; GeneTex, Irvine, CA, USA), anti-lamin B1 (12987-1-AP, 1:2000; Proteintech, Rosemont, IL, USA), anti-HO-1 (43966S, 1:2000; Cell Signaling, Danvers, MA, USA), anti-NQO1 (ab34173, 1:5000; Abcam, Cambridge, UK), anti-GCLC (ab207777, 1:2000; Abcam), and anti-GCLM (ab153967, 1:1000; Abcam). Horseradish peroxidase-conjugated secondary antibodies (AP132P, 1:4000; Merck Millipore, Darmstadt, Germany) were used. Immunoblots were detected using a chemiluminescence kit (Merck Millipore). 

### 2.4. Immunocytochemistry

C6 cells (4 × 10^4^) were treated with Tau-Cl (200 μM) for 3 or 9 h or with taurine (200 μM) for 9 h, and then fixed with 4% paraformaldehyde for 20 min. The anti-Nrf2 antibody (16396-1-AP, 1:200; Proteintech) was incubated overnight, followed by incubation with FITC-labeled anti-IgG antibody (AP132F, 1:200; Merck Millipore). Cells were counterstained with 4′,-6-diamidino-2-phenylindole (DAPI; H-1200; Vector Laboratories, Newark, CA, USA) to visualize the nuclei. Images were observed under a Zeiss LSM 510 META microscope (Carl Zeiss Meditec AG, Jena, Germany).

### 2.5. Modification of the Thiol Groups of Keap1 by Tau-Cl

C6 cells were incubated with 200 or 500 μM Tau-Cl for 5, 10, and 20 min, and then washed three times with PBS. Thereafter, the cells were lysed with RIPA buffer containing 2 mM PMSF and 20 μg/mL chymostatin. The lysate was incubated with 1 μg/mL biotin polyethyleneoxide iodoacetamide (Biotin-IAA) (Sigma) for 18 h. The mixtures were incubated with 20 μL of streptavidin–agarose beads (50% slurry) (Pierce, Rockland, IL, USA) for 1 h at 4 °C with rotation and centrifuged at 10,000× *g* for 1 min. The pellets containing reduced Keap1 were washed three times with PBS and subjected to 10% SDS-PAGE, followed by immunoblotting with an anti-Keap1 antibody (SC-15246, 1:500; Santa Cruz Biotechnology). The supernatants containing oxidized Keap1 were incubated with 1 μg/mL biotin maleimide (Sigma) or Biotin-IAA for 18 h. Then, the mixtures were incubated with streptavidin–agarose beads, and the pellets were analyzed by immunoblotting as with reduced Keap1 samples. 

### 2.6. Reaction of the Thiol Groups of Keap1 with Tau-Cl

Titrants of thiol groups, including 2.2′-dipyridyl disulfide and 4,4′-dipyridyl disulfide, react with the thiols of Keap1 [42]. To determine the potency with which Tau-Cl reacts with thiol groups, 0.5 and 2 μM Tau-Cl or 4,4′-dipyridyl disulfide (Sigma) were added to a solution of 0.5 μM Keap1 (Sino Biological, Beijing, China) solution dissolved in PBS. Immediately after mixing, the changes in the absorbance at 325 nm were monitored for 20 min at intervals of 5 min. 

### 2.7. Cell Viability Assay

The 3-(4,5-dimethylthiazol-2-yl)-2,5-diphenyltetrazolium bromide (MTT) assay was performed to measure cell viability. C6 cells were pre-treated with 50, 100, or 200 μM of Tau-Cl or 200 μM of taurine for 9 h, followed by treatment with 300 μM of H_2_O_2_ for 1 h in DMEM containing 10% FBS. After 24 h, the cells were incubated with 1 mg/mL MTT (Sigma) for 1 h. The medium was removed and 500 μL of DMSO was added to solubilize the formazan product. The mixture (100 μL) was added to a 96-well plate and the absorbance was measured at 540 nm using a microplate reader.

### 2.8. siRNA Transfection

C6 cells (4 × 10^4^) were seeded in 24-well plates at 24 h before transfection. Rat HO-1-specific siRNA (siHO-1; 5′-GUC AUG GCC ACU UUG AUA UCA GUG T-3′ and 5′-ACA CUG AUA UCA AAG UGG CCA UGA CGC-3′) and a nonspecific siRNA (siCon; 5′-CGU UAA UCG CGU AUA AUA CGC GUA T-3′ and 5′-AUA CGC GUA UUA UAC GCG AUU AAC GAC-3′) were purchased from Integrated DNA Technologies, Inc. (Coralville, IA, USA). The siHO-1 or siCon (both 40 pM) was mixed with 1 μL/well of Oligofectamine reagent (Invitrogen, Carlsbad, CA, USA) in Opti-MEM (Invitrogen) according to the manufacturer’s instructions. The siRNA-Oligofectamine complexes were added to C6 cells. After incubation for 15 h, the cells were treated with 200 μM Tau-Cl.

### 2.9. Reactive Oxygen Species (ROS) Quantification 

C6 cells (4 × 10^4^) were seeded into 24-well plates and cultured for 24 h. C6 cells were pre-treated with 200 μM of Tau-Cl or 200 μM of taurine for 9 h, followed by treatment with 300 μM of H_2_O_2_ for 1 h. Cells were then incubated in DMEM containing 10 μM 5-(and-6)-chloromethyl-2′,7′-dichlorodihydrofluorescein diacetate (CM-H_2_DCFDA; Thermo Fisher Scientific, Waltham, MA, USA) for 30 min. After washing cells with PBS, fluorescence and differential interference contrast images were obtained using a Zeiss microscope. Quantified fluorescence changes were obtained using ImageJ (http://rsbweb.nih.gov/ij/). 

### 2.10. Statistical Analysis

The statistical analyses were performed using analysis of variance (ANOVA) followed by the Newman–Keuls test. Results are presented as the mean ± standard error of the mean (SEM). Statistical significance was accepted for *p* value < 0.05. The analyses were performed using PRISM software 5.0 (Graph Pad Software, Boston, MA, USA). 

## 3. Results

### 3.1. Tau-Cl Enhances the Expression and Nuclear Translocation of Nrf2 in C6 Cells

**Figure 1:**
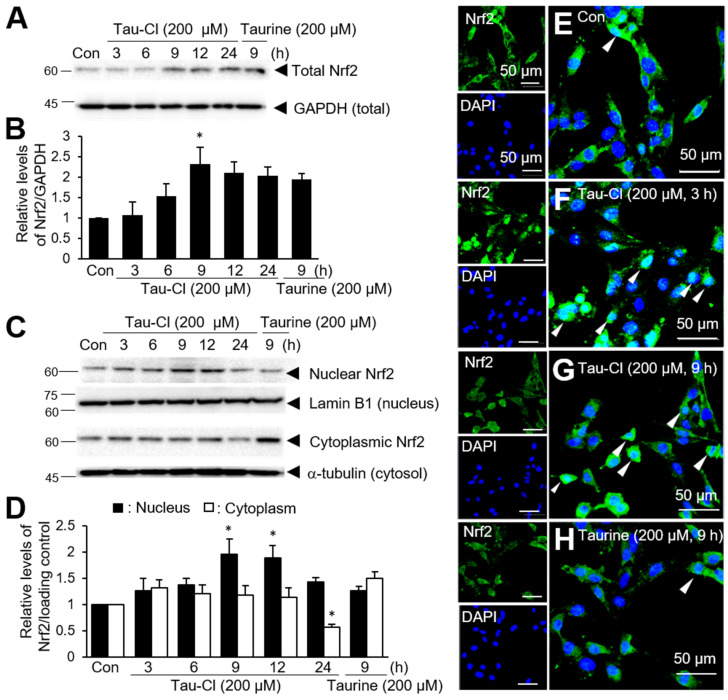
The upregulation and cytoplasmic-to-nuclear translocation of Nrf2 by Tau-Cl in C6 cells. C6 cells were treated with 200 μM of Tau-Cl for 3, 6, 9, 12, or 24 h or with 200 μM of taurine for 9 h. Nrf2 levels in total cell lysates (**A**,**B**) or in nuclear and cytosolic fractions (**C**,**D**) were determined by immunoblotting. Representative images are presented in A and C, and quantified results are presented as mean ± SEM (n = 3) in (**B**,**D**). (**E**–**H**) C6 cells were treated with 200 μM of Tau-Cl for 3 or 9 h or with 200 μM of taurine for 9 h. Double immunofluorescence staining was performed using anti-Nrf2 antibody and DAPI. Arrowheads indicate Nrf2 in the nucleus. The scale bar represents 50 μm. * *p* < 0.05 versus untreated controls.

To investigate whether Tau-Cl induces Nrf2 activation in C6 cells, the cells were treated with 200 μM Tau-Cl for 3, 6, 9, 12, or 24 h. The total amount of Nrf2 gradually increased and reached the maximum level after 9 h of treatment (Figure 1A,B). The elevated Nrf2 levels persisted until 24 h (Figure 1A,B). Next, we investigated whether Tau-Cl induces Nrf2 translocation from the cytoplasm to the nucleus. Nuclear Nrf2 levels significantly increased after 9 h of Tau-Cl treatment and the enhanced level was maintained until 12 h (Figure 1C,D); in contrast, cytoplasmic Nrf2 levels gradually decreased during the same period (Figure 1C,D). In the case of taurine (200 μM for 9 h), while total Nrf2 levels moderately increased (Figure 1A,B), the nuclear translocation of Nrf2 remained inconclusive (Figure 1C,D). To confirm the translocation of Nrf2 from the cytoplasm to the nucleus by Tau-Cl, double immunofluorescence staining was performed using an anti-Nrf2 antibody and DAPI. Under normal conditions, Nrf2 was detected in the cytoplasm of C6 cells (Figure 1E). However, it was detected in the nucleus as early as 3 h after Tau-Cl treatment, with a significant increase in nuclear accumulation at 9 h, when co-localization of Nrf2 and DAPI was evident (Figure 1F,G). Consistent with the results obtained from immunoblotting, the nuclear translocation of Nrf2 was not evident after taurine treatment (200 μM for 9 h) (Figure 1H). Together, these results suggest that Tau-Cl increases the total amount of Nrf2 and induces its translocation from the cytoplasm to the nucleus in astrocytes.

### 3.2. Tau-Cl Induces the Upregulation of Various Antioxidant Enzymes Downstream of Nrf2 

**Figure 2:**
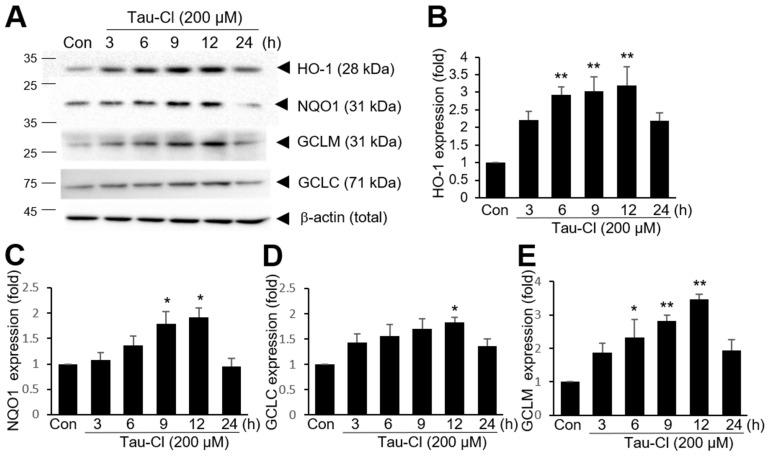
Induction of various antioxidant genes by Tau-Cl in C6 cells. C6 cells were treated with Tau-Cl (200 μM) for 3, 6, 9, 12, or 24 h and protein levels of HO-1, NQO1, GCLM, GCLC, and β-actin were determined by immunoblotting. Representative images of the protein levels are in (**A**) and quantified results are presented in (**B**–**E**) as mean ± SEM (n = 3). * *p* < 0.05 and ** <0.01 versus untreated controls.

Because Nrf2 induces the expression of various antioxidant genes, we investigated whether Tau-Cl upregulates the expression of antioxidant genes in C6 cells. C6 cells were treated with 200 μM Tau-Cl for 3, 6, 9, 12, or 24 h. HO-1 expression significantly increased after 6 h of 200 μM Tau-Cl treatment, reached the maximum level at 12 h, and subsequently decreased (Figure 2A,B). Similarly, Tau-Cl significantly induced NQO1, GCLC, and GCLM in C6 cells; however, the timing of induction differed for each gene (Figure 2). Collectively, these results suggest that Tau-Cl activates Nrf2 and induces the upregulation of various antioxidant genes in astrocytes.

### 3.3. Tau-Cl Induces the Disulfide Bond Formation of Keap1

**Figure 3:**
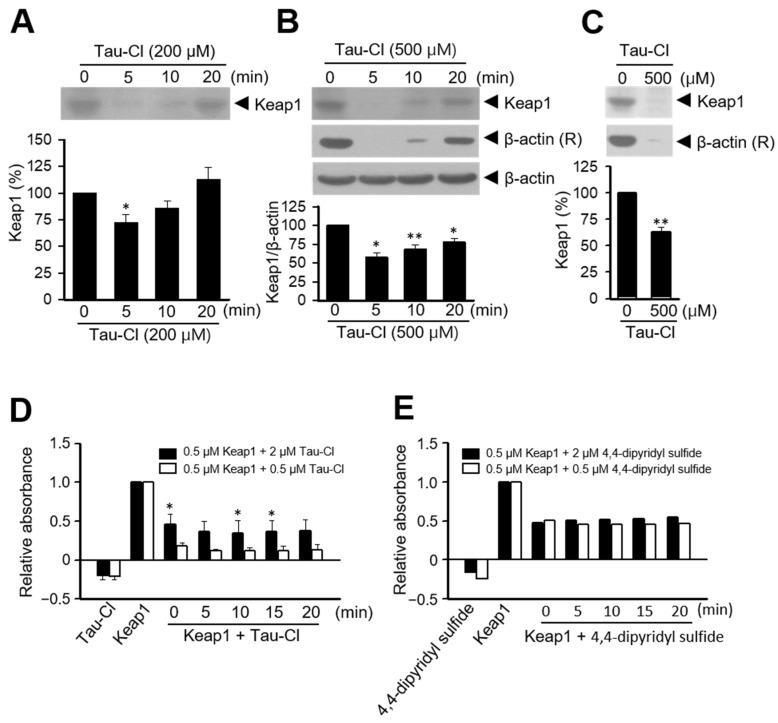
Modification of the thiol groups of Keap1 by Tau-Cl. (**A**–**C**) C6 cells were incubated with 200 or 500 μM Tau-Cl and the cell lysates were reacted with Biotin-iodoacetamide (IAA). IAA-bound Keap1 was precipitated, subjected to SDS-PAGE, and determined by immunoblotting with anti-Keap1 antibody. The quantified results are presented as mean ± SEM (n = 5 for (**A**,**B**), n = 4 for (**B**)). * *p* < 0.05 and ** <0.01 versus untreated controls. (**D**,**E**) 0.5 and 2 μM Tau-Cl (**D**) or 4,4′-dipyridyl disulfide (**E**) were added to 0.5 μM Keap1 solution, and the changes in the absorbance at 325 nm were monitored for 20 min at intervals of 5 min. The quantified results are presented as mean ± SEM (n = 3 for (**D**) and n = 1 for (**E**)). * *p* < 0.05 versus 0.5 μM Keap1.

Keap1 binding leads to the anchoring of Nrf2 in the cytoplasm, which is attached to the actin cytoskeleton. Inducers and electrophiles disrupt the Keap1-Nrf2 complex, and Nrf2 translocates to the nucleus, where it binds to the ARE region and initiates transcription [42]. Because Tau-Cl activates Nrf2 and increases the expression of downstream genes including HO-1, NQO-1, and GCLM (Figure 1 and Figure 2), we elucidated whether Tau-Cl disrupts the binding between Keap1 and Nrf2. Maleimide and iodoacetamide (IAA) are known to bind to free thiol (-SH) groups [43]. Tau-Cl treatment decreased IAA-bound Keap1 at 5 and 10 min (Figure 3A,B), suggesting that Tau-Cl increases the oxidation of the thiol groups of Keap1 to generate disulfide bonds. Next, we attempted to identify oxidized Keap1; however, it remained undetectable within our experimental system. In addition, Tau-Cl decreased IAA-bound beta-actin (Figure 3B,C; β-actin (R)), suggesting its ability to oxidize thiol groups.

The thiol groups of Keap1 react with 4,4′-dipyridyl disulfide and reveal distinct UV absorptions; therefore, 4,4′-dipyridyl disulfide is used as a spectroscopic titration reagent for the thiol groups [42]. The addition of Tau-Cl to Keap1 solution decreased its UV absorption at 325 nm in a manner similar to 4,4′-dipyridyl disulfide (Figure 3D,E), suggesting that Tau-Cl modifies the thiol groups of Keap1. Nevertheless, this method has experimental limitations owing to the difficulties associated with obtaining a sufficiently high concentration of Keap1 to reveal significant absorbance changes. Moreover, Keap1 undergoes rapid oxidation under normal laboratory conditions.

### 3.4. Tau-Cl Suppresses H_2_O_2_-Induced Cell Death in C6 Cells

**Figure 4:**
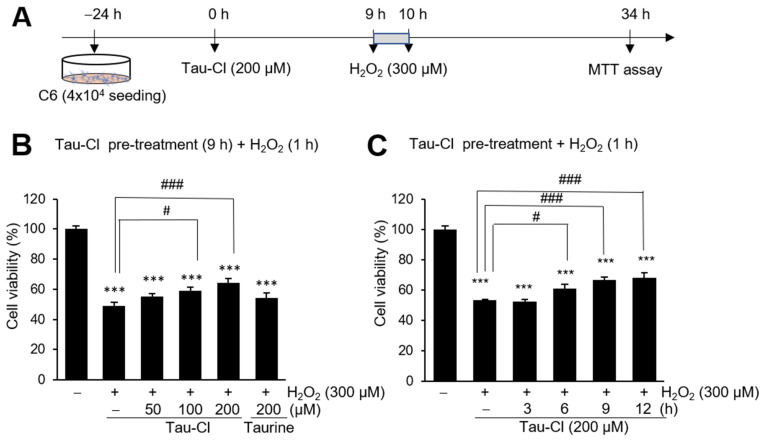
Protection of H_2_O_2_-treated C6 cells by Tau-Cl. (**A**) Schematic diagram of time points for treatment of Tau-Cl and H_2_O_2_ and cell viability assay. (**B**) C6 cells were pre-treated with Tau-Cl (50, 100, or 200 μM) or taurine (200 μM) for 9 h and then treated with H_2_O_2_ (300 μM) for 1 h. (**C**) C6 cells were pre-treated with 200 μM of Tau-Cl for 3, 6, 9, or 12 h and then treated with H_2_O_2_ (300 μM) for 1 h. For all experiments, MTT assays were performed 24 h after H_2_O_2_ treatment and cell viabilities are presented as mean ± SEM (n = 8). *** *p* < 0.001 versus untreated controls, ^#^ *p* < 0.05 and ^###^ *p* < 0.001 between indicated groups.

To determine whether Tau-Cl confers protective effects in C6 cells, we examined the cell viability of C6 cells after H_2_O_2_ treatment in the presence or absence of Tau-Cl (Figure 4A). The viability of C6 cells was decreased to 49.0 ± 2.2% after treatment with 300 μM H_2_O_2_ for 1 h (Figure 4B). However, the pretreatment of C6 cells with 100 and 200 μM Tau-Cl for 9 h significantly improved cell survival to 59.2 ± 2.0% and 64.2 ± 3.0%, respectively (Figure 4B). In contrast, pretreatment with 200 μM taurine for 9 h did not improve the cell viability (Figure 4B). Moreover, cell survival was increased after treatment of 200 μM Tau-Cl for 6, 9, and 12 h (Figure 4C). These findings suggest that Tau-Cl inhibits astrocyte cell death induced by H_2_O_2_. 

### 3.5. Tau-Cl-Mediated HO-1 Induction Is Responsible for the Protective Effects in C6 Cells

**Figure 5:**
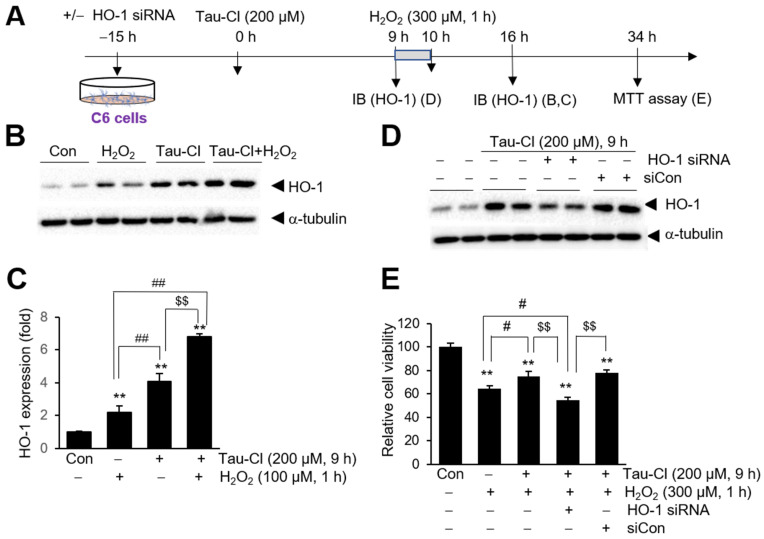
Suppression of Tau-Cl-mediated protective effects in H_2_O_2_-treated C6 cells by HO-1 knockdown. (**A**) Schematic diagram of time points for HO-1 knockdown, treatment of Tau-Cl and H_2_O_2_, and cell viability assay. (**B**,**C**) C6 cells were pre-treated with Tau-Cl (200 μM) for 9 h and then treated with H_2_O_2_ (100 μM) for 1 h. Protein levels of HO-1 were determined by immunoblotting at 6 h after H_2_O_2_ treatment. (**D**) C6 cells were transfected with HO-1 siRNA or non-specific siRNA (siCon). After 15 h, cells were treated with Tau-Cl (200 μM) for 9 h. HO-1 levels were determined by immunoblotting. Representative images of the protein levels are in B and D and quantified results are presented D as mean ± SEM (n = 3). (**E**) Cell viabilities of C6 cells after Tau-Cl pretreatment/H_2_O_2_ treatment with and without HO-1 siRNA transfection were examined using MTT assays at 24 h after H_2_O_2_ treatment. Results are presented as mean ± SEM (n = 3). ** *p* < 0.01 versus untreated controls, ^##^ *p* < 0.01, ^#^ *p* < 0.05 and ^$$^ *p* < 0.01 between indicated groups.

We examined whether the Tau-Cl-mediated protective effect is related to the upregulation of antioxidant genes in C6 cells, particularly HO-1, using siRNA-mediated HO-1 knockdown (Figure 5A). HO-1 was significantly induced in C6 cells after H_2_O_2_ treatment (100 μM, 1 h) (2.2-fold) (Figure 5B,C). Furthermore, preincubation with 200 μM Tau-Cl for 9 h further enhanced H_2_O_2_-induced HO-1 upregulation by 6.8-fold (Figure 5B,C), indicating that Tau-Cl augmented H_2_O_2_-induced HO-1 upregulation. Moreover, Tau-Cl preincubation enhanced H_2_O_2_-induced Nrf2 expression and other Nrf2-regulated antioxidant enzymes such as GCLM, GCLC, and NQO1 (Figure S1). When C6 cells were transfected with HO-1 siRNA, Tau-Cl-induced HO-1 expression decreased to 43.5 ± 0.3% compared with siRNA-non-transfected control cells (Figure 5D). However, this reduction was not observed in cells transfected with nonspecific siRNA (siCon) (Figure 5D). Importantly, the increased viability observed for Tau-Cl-pretreated/H_2_O_2_-treated cells was significantly reduced in HO-1 siRNA-transfected cells, that is, it decreased from 75.1 ± 4.2% to 54.7 ± 2.4% (Figure 5E). However, a similar level of protective effect was observed in siCon-transfected cells (Figure 5E), indicating that HO-1 is responsible for the protective effect of Tau-Cl. Taken together, these results suggest that Tau-Cl-mediated HO-1 upregulation is responsible for the protective effects of Tau-Cl in H_2_O_2_-treated astrocytes.

### 3.6. Tau-Cl Inhibits ROS Production in H_2_O_2_-Treated C6 Cells

**Figure 6:**
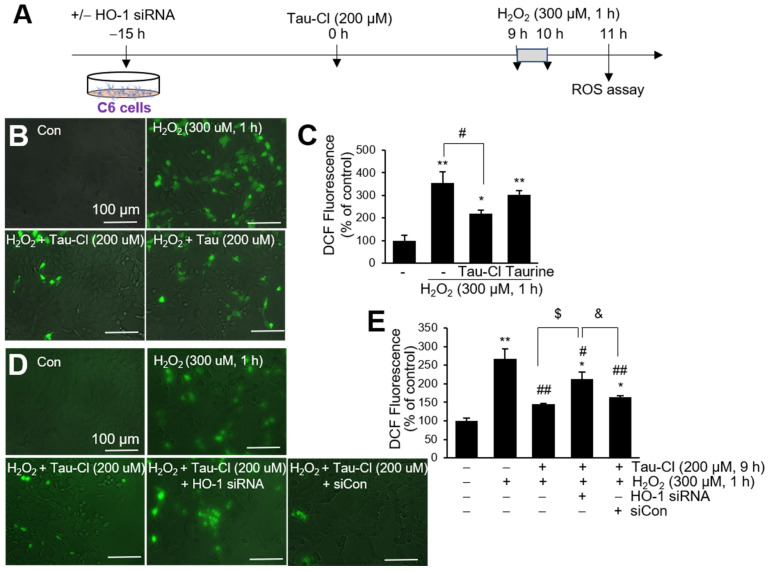
Suppression of H_2_O_2_-induced ROS production by Tau-Cl in C6 cells. (**A**) Schematic diagram of time points for HO-1 knockdown, treatment of Tau-Cl and H_2_O_2_, and ROS assay. (**B**,**C**) C6 cells were pre-treated with Tau-Cl (200 μM) for 9 h and then treated with H_2_O_2_ (300 μM) for 1 h. Intracellular ROS levels were measured using CM-H_2_DCFDA at 1 h after H_2_O_2_ treatment. (**D**,**E**) C6 cells were transfected with HO-1 siRNA or non-specific siRNA (siCon), pre-treated with Tau-Cl (200 μM) for 9 h, and then treated with H_2_O_2_ (300 μM) for 1 h. ROS levels in C6 cells with and without HO-1 siRNA transfection were examined using CM-H_2_DCFDA at 1 h after H_2_O_2_ treatment. Results are presented as mean ± SEM (n = 3). The scale bar represents 100 μm. ** *p* < 0.01, * *p* < 0.05 versus untreated controls, ^#^ *p* < 0.05 and ^##^ *p* < 0.01 versus H_2_O_2_ controls, ^$^ *p* < 0.05 and ^&^ *p* < 0.05 between indicated groups.

We then investigated whether Tau-Cl suppresses ROS induction in H_2_O_2_-treated C6 cells (Figure 6A). When C6 cells were treated with H_2_O_2_ (300 μM, 1 h) and stained with CM-H_2_DCFDA, an intracellular ROS indicator, induction of DCF fluorescence was observed (Figure 6B,C). Interestingly, pretreatment of C6 cells with 200 μM of Tau-Cl for 9 h significantly suppressed the induction of DCF to 69.8 ± 2.9% of that in treatment-naïve H_2_O_2_ control cells (Figure 6B,C). In contrast, pretreatment with 200 μM of taurine for 9 h failed to suppress DCF induction (Figure 6B,C), demonstrating that Tau-Cl suppressed H_2_O_2_-induced ROS generation in H_2_O_2_-treated C6 cells. Importantly, suppression of ROS induction by Tau-Cl was not detected in HO-1 siRNA-transfected C6 cells, however, ROS induction was suppressed in siCon-transfected C6 cells (Figure 6D,E), suggesting that HO-1 is responsible for this suppression. Taken together, these results indicate that Tau-Cl suppresses ROS induction in H_2_O_2_-treated C6 cells and HO-1 upregulation is responsible for this effect.

## 4. Discussion

Astrocytes exert protective effects on neurons via their antioxidant activity [44]. They produce antioxidant molecules in the brain, playing a vital role in preventing ROS elevation and neuronal cell death in various CNS disorders [45]. HO-1 expression is weak in the brain and is limited to small groups of neurons and neuroglia [46]. However, the protective effects of HO-1 in the brain have been reported under several pathological conditions [47,48,49]. In an animal model of intracerebral hemorrhage, the strong neuroprotective effects of HO-1 overexpression in astrocytes have been reported [50]. In the present study, we demonstrated the autocrine function of Tau-Cl-mediated HO-1 induction, i.e., its ability to suppress H_2_O_2_-induced astrocyte cell death. However, astrocyte HO-1 may confer paracrine effects on neighboring cells, including neurons and microglia. In previous studies, we demonstrated the neuroprotective effect of HO-1 in astrocytes using conditioned media [47,48] and in animal models of MCAO [49]. Recently, Zhang et al. [51] reported that astrocyte-derived exosomes protect hippocampal neurons after traumatic brain injury by activating Nrf2 signaling in both rat and mouse models. In addition to neurons, studies have reported the beneficial effects of astrocyte HO-1 on microvascular function after various acute injuries, including post-ischemic myocardial injury [52], hemorrhagic shock [53], and seizures [54]. Alfieri et al. [55] reported that preconditioning stimuli increase HO-1 expression, primarily in perivascular astrocytes, which are responsible for preserving the barrier function in a transient rat MCAO model. Nevertheless, additional studies are warranted to investigate the protective effects of enhanced astrocyte HO-1 levels in other brain cell types.

Taurine is one of the most abundant free amino acids in the brain. However, its concentration in the brain decreases with age. Astrocytes are the primary taurine producers in the CNS [56]. Astrocytes release taurine as a gliotransmitter and provide neurons with hypotaurine as a substrate for taurine production [56]. As mentioned above, astrocytes play vital roles in maintaining normal brain function as well as protecting against inflammatory responses in the brain. Reactive astrocytes release inflammatory cytokines, produce various antioxidant molecules, including GSH, and activate ROS-detoxifying enzymes such as GST, GSH peroxidase, thioredoxin reductase, and catalase to improve neuronal survival [57,58,59]. In our previous study, we observed that neutrophils infiltrate into the ischemic region and that Tau-Cl exerts neuroprotective effects in the post-ischemic brain after MCAO and BV2 cells via increasing the levels of antioxidant enzymes, including HO-1 [20]. In the present study, we elucidated the effects of Tau-Cl in astrocytes. Tau-Cl-induced upregulation and nuclear translocation of Nrf2 as well as upregulated Nrf2-regulated antioxidant genes (Figure 1 and Figure 2), suggesting that Tau-Cl-derived antioxidants protect astrocytes and ameliorate neuronal injury. Tau-Cl recovered cell survival against oxidative stress (H_2_O_2_)-induced cell death (Figure 4), which was associated with increased HO-1 expression and decreased ROS production (Figure 5 and Figure 6). Therefore, the induction of HO-1 by Tau-Cl mitigates H_2_O_2_-induced astrocyte cell death through its anti-oxidative properties. 

Under homeostatic conditions, the levels of Nrf2 protein are maintained at a relatively low level owing to constitutive ubiquitin-mediated proteasomal degradation of Nrf2 by Keap1 [27,60]. Nrf2 is primarily localized in the cytoplasm with interaction with Keap1. In response to oxidative stress or electrophilic attacks, Keap1 undergoes oxidation at specific cysteine residues, leading to the disruption of its interaction with Nrf2. The disruption results in the stabilization of Nrf2, a critical step in initiating its translocation into the nucleus to regulate transcriptional activities. The translocation of Nrf2 into the nucleus occurs by interacting with the importins, facilitated by the three nuclear localization sequences (NLS) located in Neh1, Neh2, and Neh3 domains [61,62]. Importins recognize the NLS of Nrf2, allowing Nrf2-importin complexes to traverse the nuclear pore complex, comprising various nucleoporins that regulate the transport of molecules between the cytoplasm and the nucleus. 

There are two major mechanisms by which Nrf2 dissociates from Keap1: one is a conformational change in Keap1 and the other is the phosphorylation of the serine residues of Nrf2 [63,64]. For the phosphorylation of the serine residues of Nrf2, various protein kinases, particularly mitogen-activated protein kinases (MAPKs), can trigger transcriptional activity via Nrf2 phosphorylation. We observed that Tau-Cl exhibited no stimulatory effects on various protein kinases, including MAPKs. Moreover, Tau-Cl inhibited lipopolysaccharide-induced MAPK activation in RAW 264.7 cells [41]. Therefore, we determined the effect of Tau-Cl on the conformational changes of Keap1, with particular attention on determining whether Tau-Cl modifies the reactive cysteine residues of Keap1, which play a pivotal role in its association with Nrf2. Human Keap1 contains 27 cysteines with thiol groups that form covalent bonds with other cysteine thiols, bind to metals and metalloids, and react with oxidants [65]. The chemical modification of the cysteine residues of Keap1 results in the loss of Nrf2 repressor function and changes the expression of Nrf2 target genes, which collectively restore redox balance and resolve inflammation, thereby ensuring a comprehensive cytoprotection. Tau-Cl decreased IAA-bound Keap1 (Figure 3), suggesting that Tau-Cl converts the thiol groups into disulfide bonds. Most Nrf2 inducers are electrophiles that readily react with the cysteine thiol groups of Keap1 [42]. As a titrant for thiols, 4,4′-dipyridyl disulfide exhibits unique UV spectra; its absorbance changes when it reacts with thiols. Tau-Cl decreased the UV absorption of Keap1 similarly to 4,4′-dipyridyl disulfide, suggesting that Tau-Cl changes the thiol groups of Keap1 (Figure 3). Although we could not identify the cysteine residues targeted by Tau-Cl, we provided tentative evidence that Tau-Cl changes the thiol groups of Keap1.

## 5. Conclusions

In the present study, we investigated the antioxidative effects of Tau-Cl in astrocytes. Tau-Cl augmented Nrf2 expression and activation and upregulated Nrf2-regulated antioxidants such as HO-1, NQO1, GCLC, and GCLM. The activation of Nrf2 by Tau-Cl occurs by disrupting the Keap1-Nrf2 complex via the oxidation of the thiol groups of Keap1. Consequently, Tau-Cl rescued the cells from H_2_O_2_-induced death by further enhancing HO-1 expression and suppressing ROS production. Nevertheless, additional studies are required to comprehensively understand the role of Tau-Cl not only in astrocytes but also in other glial cells and neurons. The neuroprotective effects of Tau-Cl in astrocytes can be a promising therapeutic target for various neuroinflammatory and neurodegenerative diseases. 

## Data Availability

The data presented in this study are included in the article and are also available on request from the corresponding authors.

## Supplementary Materials

The following supporting information can be downloaded at: https://www.mdpi.com/article/10.3390/antiox13020169/s1, Figure S1: Up-regulations of genes downstream of Nrf2 by Tau-Cl-pre-treatment in H_2_O_2_-treated C6 cells.
