# Supplementary material for: Taurine Chloramine-Mediated Nrf2 Activation and HO-1 Induction Confer Protective Effects in Astrocytes
Source: Antioxidants (Basel). 2024 Jan 29;13(2):169. doi: 10.3390/antiox13020169 (PMC10886344; doi:10.3390/antiox13020169)
Supplement: Supplementary file 1 [file antioxidants-13-00169-s001.zip › antioxidants-2802628-supplementary.pdf]

Supplementary Figure S1

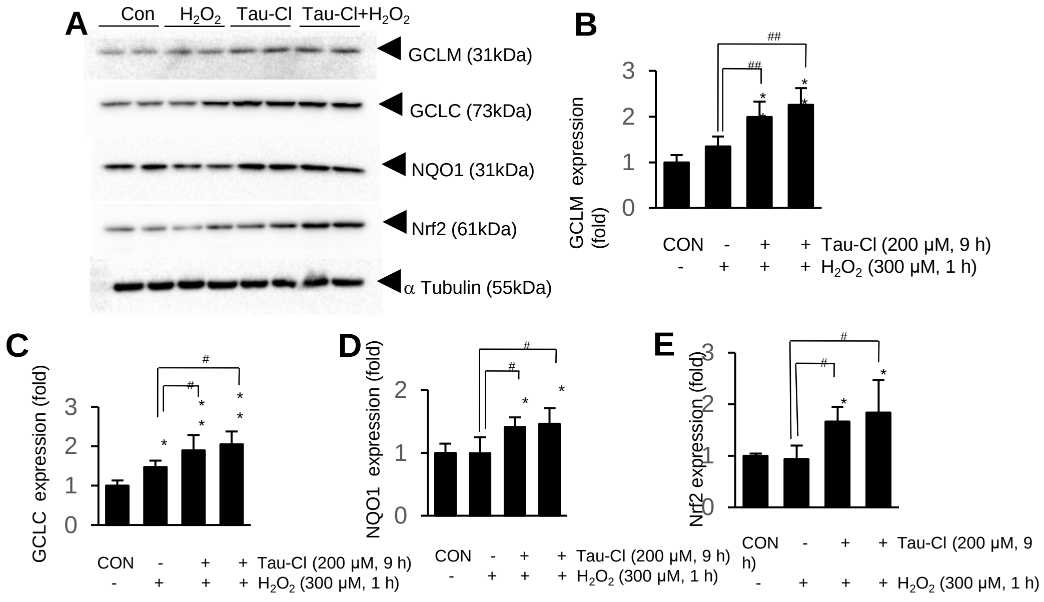

Supplementary Figure S1. Up-regulations of genes downstream of Nrf2 by Tau-Cl-pre-treatment in H_2O_2-treated C6 cells.

C6 cells were pre-treated with 200 μM of Tau-Cl for 9 h, and then treated with H_2O_2 (300 μM) for 1 h. Levels of GCLM, GCLC, NQO1, and Nrf2 were determined at 6 h after H_2O_2 treatment. Representative images are presented in A and quantified results are presented in B-E as mean ± SEM (n=4). *p<0.05 and **p<0.01 versus untreated controls, #p<0.05 and ##p<0.01 between indicated groups.
